# A hybrid soft material robotic end-effector for reversible in-space assembly of strut components

**DOI:** 10.3389/frobt.2023.1099297

**Published:** 2023-06-26

**Authors:** Maxwell Hammond, Anthony Dempsey, William Ward, Stephen Stewart, James H. Neilan, Jessica Friz, Caterina Lamuta, Venanzio Cichella

**Affiliations:** ^1^ Department of Mechanical Engineering, University of Iowa, Iowa City, IA, United States; ^2^ Department of Mechanical Engineering, Clemson University, Clemson, SC, United States; ^3^ Department of Physics and Astronomy, University of Central Arkansas, Conway, AR, United States; ^4^ Department of Mechanical Engineering, North Carolina State University, Raleigh, NC, United States; ^5^ Space Technology and Exploration Directorate, Langley Research Center, NASA, Hampton, VA, United States; ^6^ Simulation Development and Analysis Branch, Langley Research Center, NASA, Hampton, VA, United States

**Keywords:** autonomous robotic assembly, in-space assembly, modular manipulator, soft material robotics, soft robotics

## Abstract

Based on the NASA in-Space Assembled Telescope (iSAT) study (Bulletin of the American Astronomical Society, 2019, 51, 50) which details the design and requirements for a 20-m parabolic in-space telescope, NASA Langley Research Center (LaRC) has been developing structural and robotic solutions to address the needs of building larger in-space assets. One of the structural methods studied involves stackable and collapsible modular solutions to address launch vehicle volume constraints. This solution uses a packing method that stacks struts in a dixie-cup like manner and a chemical composite bonding technique that reduces weight of the structure, adds strength, and offers the ability to de-bond the components for structural modifications. We present in this paper work towards a soft material robot end-effector, capable of suppling the manipulability, pressure, and temperature requirements for the bonding/de-bonding of these conical structural components. This work is done to investigate the feasibility of a hybrid soft robotic end-effector actuated by Twisted and Coiled Artificial Muscles (TCAMs) for in-space assembly tasks. TCAMs are a class of actuator which have garnered significant recent research interest due to their allowance for high force to weight ratio when compared to other popular methods of actuation within the field of soft robotics, and a muscle-tendon actuation design using TCAMs leads to a compact and lightweight system with controllable and tunable behavior. In addition to the muscle-tendon design, this paper also details the early investigation of an induction system for adhesive bonding/de-bonding and the sensors used for benchtop design and testing. Additionally, we discuss the viability of Robotic Operating System 2 (ROS2) and Gazebo modeling environments for soft robotics as they pertain to larger simulation efforts at LaRC. We show real world test results against simulation results for a method which divides the soft, continuous material of the end-effector into discrete links connected by spring-like joints.

## 1 Introduction

### 1.1 In-space assembly

Advancing human space exploration entails developing larger and more sustainable structures in space and on other worlds. This feat requires in-space servicing, assembly, and manufacturing (ISAM). Identified as the next strategic thrust for the National Aeronautics and Space Administration (NASA), in-space assembly (ISA) offers key possibilities by freeing a mission from the current restrictions of mass and volume on launch vehicles. In this field, it is crucial to consider optimization of assembly agents and methods designed to enable diverse applications while minimizing launch costs. This can be achieved by pursuing novel, purpose-built structures for specific missions. To this end, ISA researchers have been developing various technology capabilities required to make larger in-space assembled assets. For example, in 2002, LaRC’s Automated Telescope Assembly Lab (ASAL) autonomously assembled and disassembled an 8-m truss structure (Doggett, 2002). Recently, research in in-space assembly has increased with NASA funding technology development for three tipping point In-Space Robotic Manufacturing and Assembly (IRMA) projects, Dragonfly (Lymer, 2018), Archinaut (Patané et al., 2017), and the Commercial Infrastructure for Robotic Assembly and Servicing (CIRAS) (Bowman et al., 2018). In Addition, the NASA iSAT Study recommended to the 2020 Astronomy and Astrophysics Decadal Survey that ISAM be considered as an enabling method for future large space telescopes for its risk, cost, and science benefits (Mukherjee et al., 2019).

### 1.2 Precision Assembled Space Structures

The iSAT study (Mukherjee et al., 2019) examined methods and technologies for efficiently creating large-diameter telescopes. A concern noted in the study was the difficulty associated with assembling a precision mirror backing structure for such a telescope. The NASA LaRC Precision Assembled Space Structure (PASS) project in the Space Technology Mission Directorate (STMD) Game Changing Development (GCD) Program seeks to retire this risk by autonomously assembling a 20-m diameter telescope mirror backbone structure in a laboratory environment (Doggett et al., 2022). Some of the areas that PASS is investigating to accomplish this are the structural components and their storage. One solution is the nested tapered column strut (Bush and Mikulas, 1976b). This strut design uses a stowed stacked configuration akin to stacked dixie cups and allows for more struts to be stored *versus* full length units. It has been shown in Bush and Mikulas (1976a) that non-tapered cylindrical columns result in volume limited payloads on the previous Space Shuttle and current launch volumes, but nestable tapered columns easily eliminate this problem. In order to use this strut type, PASS has also researched methods of joining composite materials of this design. In doing so, the ability to bond and even de-bond conical strut segments was identified as a priority for the project. This priority requires an end-effector capable of grasping conical surfaces and applying both heat and pressure to join the strut pieces with a selected adhesive. Out of this requirement, we propose a design framework for a soft material robot end-effector capable of producing the required pressure and temperature for the bonding/de-bonding of structural components and conduct small scale tests on the components of this design for the purpose of validation.

### 1.3 Soft robotics

Inherent compliance in soft robotic systems offers unique actuation and resilience to impact damage as well as reduced risk to task spaces. Within the unstructured environments often encountered in ISA, these characteristics are crucial, allowing for decision making with an incomplete information set and a larger margin for error. This decreases risk and increases the systems capability with respect to exploring unknown environments. Thus far, NASA funded research in the area of in-space soft robotics has primarily focused on mobility for exploration, and muscular assistance and human space suit augmentation. To expand research efforts into the ISA realm, LaRC has been funding soft material robotics research via the STMD Center Innovation Fund/Internal Research And Development program. We believe that soft material robotics have much to offer ISA and the use of such systems is supported by research from other organizations in the recent past.

Examples of soft material or hybrid soft/hard material robotics for space exploration include Yale University’s TT-3 (Chen et al., 2017) and the Jet Propulsion Laboratory’s (JPL) tumbleweed ball (Jones and Wu, 1999) which have used the concept of tensegrity and inflatables respectively as novel methods of traversing difficult and unknown surface terrains. Additionally, Omniskins (Booth et al., 2018) are adaptable skins that can be wrapped around various objects (e.g., Rocks) giving them the potential for mobility. For liquid environments such as Jupiter’s Moon Europa, which is believed to host water oceans under its surface, Cornell University has designed a squid like robot referred to as Roboeel (Peck, 2016). Additionally, since human space suits stiff mobility significantly increase fatigue and limit extravehicular activity duration, researchers have been working on augmenting human motion with soft actuators inside a suit. These approaches use shape memory alloys as additional tendons embedded in a suit to assist in joint mobility (Holschuh and Newman, 2016). Other efforts have also been made to investigate the use of soft robots to build structures on the surface of Mars (Lloyd, 2019).

Outside of the efforts to apply soft robotics for in-space applications, the field has significantly expanded in other directions over the last two decades (Laschi et al., 2016; Bao et al., 2018). Even in the narrower scope of grasping devices this remains true (Shintake et al., 2018). As classical pneumatic and cable driven actuation systems are joined by more modern approaches made available by the development of artificial muscles (Greco et al., 2021), more specialized systems can be created. As defined in Greco et al. (2021), an artificial muscle is a single component material or device which can be reversibly actuated given a specific external input. For in-space applications where weight and volumetric constraints are strict, the ability for systems to shed the bulk of electromagnetic motors, pumps and compressors commonly associated with classical robotic actuation is very desirable. Among these actuators, twisted and coiled artificial muscles, particularly the carbon fiber/silicone rubber muscles proposed in Lamuta et al. (2018), have demonstrated very high output force to weight ratios. These muscles can be thermally actuated through joule heating, meaning they only require a voltage source to actuate and have been shown to have desirable traits for robots including grasping devices (Sun et al., 2021).

### 1.4 Modeling and simulation

Long-lead time for manufacturing of custom components, high operational costs, and limited access to full-scale testing facilities with appropriate environmental conditions makes developing large-scale ISAM technologies more challenging. This challenge can be mitigated by using high-fidelity modeling and simulation tools. Testing in a virtual environment would allow a mission developer to evaluate overall performance of full-scale servicing, assembly, and manufacturing systems in real-time under applicable environmental conditions. Many modeling and simulation capabilities exist that may be used for system analysis, but they must be improved and integrated together to increase testing fidelity for large-scale ISAM operations (Friz et al., 2022).

Complex ISAM operations will require collaboration between multiple hardware components, including robotic agents, unique end effectors, assembly structures, and metrology system hardware. Each element must be positioned well with adequate lighting and receive metrology feedback to successfully complete different tasks. With high-fidelity modeling and simulation capabilities, multiple hardware models could be integrated and tested within a simulation environment to optimize their behavior for ISAM operations. Conceptual ISAM hardware could also be modeled, tested, and compared against existing technologies to advance their capabilities.

Soft robotic systems, for example, have gone from conceptual to the sub-scale prototyping phase for complex ISAM applications; however, modeling and control of non-linear systems is challenging due to the infinite degrees of freedom a soft system may possess. Being able to accurately model soft material robotics in simulation and couple the model to a control framework is a key interest to soft robotics designers throughout industry and academia. For future integration within a more complex, multi-agent simulation, the team wants to understand the processes by which a soft system could be modeled using the robotic simulation tool Gazebo/ROS 2, a free, open-source robotic simulation commonly used across the in-space assembly community. Testing its accuracy for modeling the soft gripper will allow this team to identify what needs to be improved for future soft system modeling and collaborate more easily with multiple partners within NASA, industry, and academia. The team set up a simple hardware and simulation test using the TCAM-actuated soft gripper which will be expanded upon further in this paper.

## 2 A hybrid soft gripper for reversible bonding

Research efforts led by the PASS project (see Section 1.2) have resulted in the development of reversible bonding agents for use in the assembly and disassembly of conical struts for structures in orbit. This adhesive undergoes its bonding reaction given pressure, temperature and time based on a known functional relationship between those elements. These elements define the parameters for the design of a soft robotic end-effector capable of applying adhesives to an array of surface geometries. This end-effector takes the form of a soft gripper which must be capable of reaching and maintaining minimum suitable bonding pressure and temperature at the adhesive’s point of contact and must be able to be actuated to grasp the target.

### 2.1 Design

**FIGURE 1:**
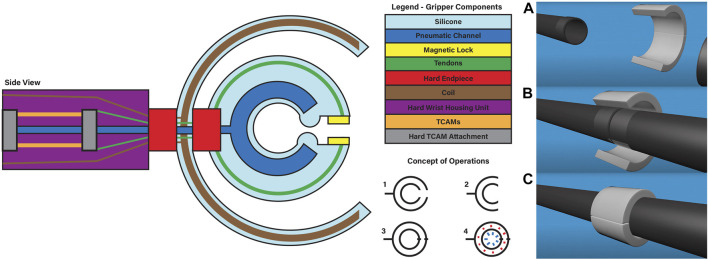
The schematic on the left gives an overview of gripper design and systems. The concept of operation steps for the gripper can be observed in the bottom center of the graphic: 1) Starting at rest. 2) The system opens, wraps around the strut. 3) The gripper locks itself around the strut. 4) The gripper applies heat and pressure to the adhesive, before reversing the steps. The image on the right shows Three-dimensional view for joining two struts together. **(A)** the struts are apart, and the gripper is away from the strut sections. **(B)** The struts are brought together, and the gripper approaches a position around the struts. **(C)** The gripper closes around the struts and then applied heat and pressure to bond the adhesive.

**FIGURE 2:**
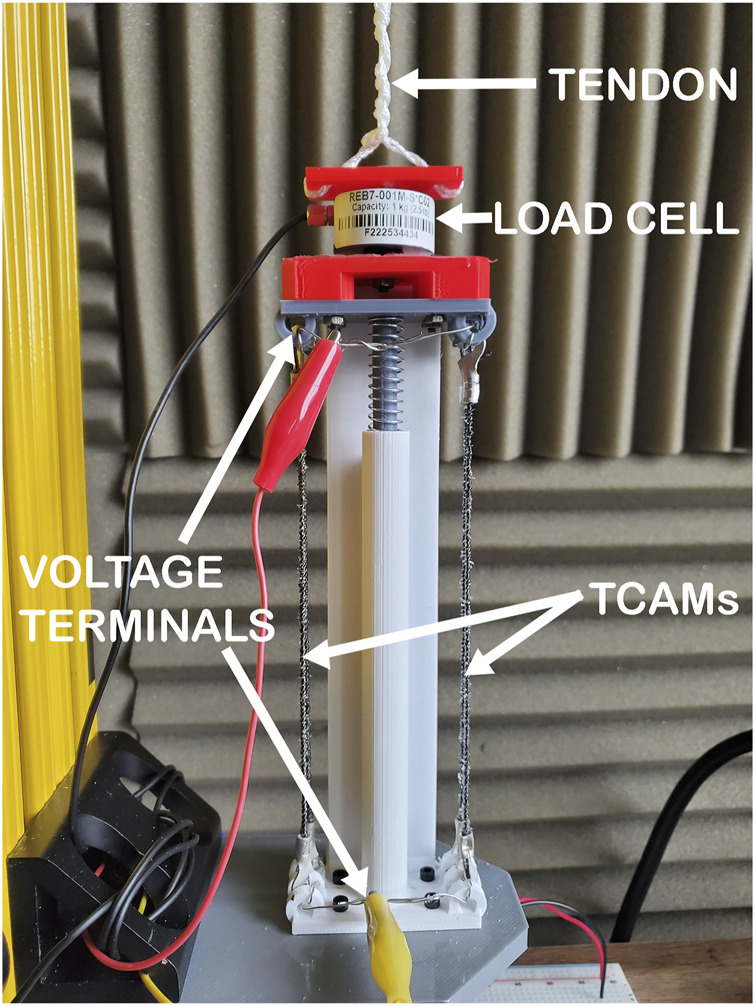
Prototype TCAM tension zeroing by spring used for testing. Here the black strands on either side are the TCAMs which form a parallel circuit with terminals at either end of the device. This actuator is attached to a load cell and the tendon extends to the gripper.

Figure 1 gives a visual overview of the systems at play within the gripper. The design can be broken down into three major subsystems: pressure, heating and actuation. Each of these three subsystems is tied to the key functional requirements for the gripper’s application to reversible bonding. It is worth noting that the manipulation of struts is handled by other systems in cooperation with the proposed gripper and they are not considered here. For the purpose of simplifying initial design, the struts are assumed to be composite cylinders, 2.54 cm in diameter and 1 m long. An adhesive covered sleeve will cover both ends of the struts when bonding them together. These sizing choices are informed by the small scale testing designs of ISA researchers developing the structures to be built with these mechanisms. Given the early stage of this work, modularity in design is important to ensure future changes easily integrated. The gripper’s pressure system is a fluid channel embedded within the molded silicone gripper, designed to direct expansion inward toward the conical struts being bonded. Heating is handled by a flexible inductive coil embedded in a soft outer enclosure around the gripper designed to induce eddy currents in the metal connection sleeve on which the target adhesive is applied. Finally, the actuation system of the gripper is handled by TCAMs held within the gripper’s wrist. Note that by observing Equation 1, it can be seen that the initial length of a TCAM is dependant on an external force acting on the muscle. As a result, TCAMs must be in tension to actuate. In order to ensure that the muscles are under constant tension without applying unwanted torque on the gripper, the wrist holding the TCAMs is designed such that muscles actuate within a closed system pulling against a spring. The closed system, seen in Figure 2, then acts as a linear actuator unit which can be connected to the tendons of the gripper. This design is modular, allowing for any number of TCAMs and any compression spring to be used in order to scale the system to larger tasks.

### 2.2 Twisted and coiled artificial muscles

In the interest of adhering to weight and volumetric constraints imposed by in-space applications, this gripper will avoid the bulk of compressors and pumps commonly found in pneumatic and hydraulic soft systems through the implementation of TCAM actuators. Progress in the field of artificial muscles have led to the recent development of carbon fiber silicone rubber (CF/SR) TCAMs which have remarkable mechanical properties. These muscles are capable of lifting 12,600 times their own weight, sustaining 60 MPa of mechanical stress and providing a tensile stroke of up to 60% while requiring only a small input of 0.2 V/cm Lamuta et al. (2018). The dynamic properties of these muscles have been well defined for the case of joule heating actuation and a brief description of the TCAM model found in Giovinco et al. (2019) is given below.

A TCAM’s motion is given by the following dynamics,

$$m\ddot{\ell }\left(t\right)+b\dot{\ell }\left(t\right)+k\left(T_{\Delta }\right)\ell \left(t\right)=F_{e}\left(t\right).$$ (1)

Here, *l*(*t*) is the length of the TCAM, *m* is the system mass, *b* is the system damping coefficient, *k*(*T*_Δ_) is the spring coefficient of the system which is expressed as a function of the system’s changing temperature, *T*_Δ_, and *F*_*e*_ is the external force acting on the muscle. The spring coefficient is expressed as,

$$k\left(T_{\Delta }\right)=\frac{\bar{m}g}{S\left[1-\frac{1}{B\left(T_{\Delta }\right)-C\left(T_{\Delta }\right)}\left(\frac{qB\left(T_{\Delta }\right)C\left(T_{\Delta }\right)}{\sqrt{B\left(T_{\Delta }\right)\bar{m}g}}-2C\left(T_{\Delta }\right)\right)\right]},$$ (2)

where

$$B\left(T_{\Delta }\right)=E_{x}\frac{\pi }{4}{\left(\text{CTE}\;T_{\Delta }\left(t\right)r_{0}+r_{0}\right)}^{4},$$

$$C\left(T_{\Delta }\right)=G_{yz}\frac{\pi }{2}{\left(\text{CTE}\;T_{\Delta }\left(t\right)r_{0}+r_{0}\right)}^{4}.$$

Here, *S* is the length of the uncoiled fiber yarn, $q=\frac{2\pi n}{S}$ is the total end rotation of the muscle, *E*_*x*_ and *G*_*yz*_ are the longitudinal Young’s and shear modulus respectively, CTE is the material linear thermal expansion coefficient, and *r*_0_ is the initial radius of the TCAM. The change in TCAM temperature for the case of joule heating is dictated by the differential equation,

$${\dot{T}}_{\Delta }\left(t\right)=\frac{V^{2}\left(t\right)}{C_{t}R}-\frac{\lambda }{C_{t}}T_{\Delta }\left(t\right),$$ (3)

Where *V* is the voltage applied to the TCAM, *R* is the electrical resistance of the TCAM, *C*_*t*_ is its thermal mass, and *λ* is its absolute thermal conductivity.

### 2.3 Reversible adhesive

A reversible adhesive, developed by ATSP Innovations, can bond to a structure and debond from a structure under certain conditions of temperature, pressure, and time (ATSP innovations, 2019). This property has sparked interest within the researchers of the PASS project as a method of bonding the nested column struts together upon arrival in orbit. A metallic sleeve with an adhesive coated inner diameter can be used as a bonding point between two struts and enable a structurally robust end product for use in the assembly of larger structures. Having the ability to configure these longer struts in-space allows more efficient packing of the struts in transport and added modularity in truss designs.

For this application, external testing of the adhesive done by the manufacturer suggests that for a successful bond or debond, the adhesive needs to heat up to a minimum temperature of 340°C and needs to undergo a minimum applied pressure of 0.5 MPa over a varying amount of time. Once the adhesive reaches these conditions, the struts can be joined together or taken apart. The soft gripper needs to ensure that the adhesive can reach these parameters for a successful mission; the selected methods to reach these parameters include induction (i.e., temperature) and TCAM actuated pneumatics (i.e., applied pressure).

### 2.4 Sensing

The gripper utilizes many variable resistance sensors which collect data during testing and provide feedback during operation. These include flex sensors for measuring the angle of deflection of the gripper arms, thermistors for measuring the temperature of the target adhesive, an analog pressure gauge to measure pneumatic pressure in testing, and a force sensitive resistor (FSR) for measuring applied pressure. An Arduino reads each sensor’s resistance and converts that resistance to useful data in real time and we use serial monitoring and MATLAB to view and analyze the data upon acquisition. The feedback from this system allows for autonomous control of the gripper and provides a way to compare its simulated operation with the real world.

Flex sensors embedded in one arm of the gripper read its angle of deflection during TCAM actuation. Flex sensors are thin, flexible substrates that increase in resistance as their bend radius decreases. Since this response is characterized by a linear relationship within the bounds of this use case, the process for their calibration is relatively simple. We hold the sensors at two different deflections, record their resistance, and use linear interpolation re-map those values to angle measurements using Arduino’s map function. Two sensors are calibrated and inserted with known position into a gripper and their measured angles are used in conjunction with a piecewise constant curvature (PCC) model (described in Section 5.1) to return the pose of the gripper’s arm during operation. The sensors used in each arm are Spectra Symbol FS-L-0055-253-ST flex sensors capable of measuring resistances from 0 to 180 degrees of deflection. The resolution of each sensor is continuous but limited to the microcontroller reading its resistance. Since Arduino’s analog input readings are returned as 10-bit integer values, the resolution is the sensing range of the flex sensor divided by 1024. For a flex sensor calibrated from 0 to 180 degrees of deflection, its resolution is 0.1758°.

An analog pressure gauge and FSRs are used to measure the pressure generated during pneumatic actuation. An FSR is a small resistor composed of multiple substrate layers that decrease in resistance as they come into contact with one another. They are a lighter and cheaper alternative to using load cells, which are more accurate but also bulkier and do not fit in the confines of the test set up. The calibration of the FSR involved placing various masses onto a known area on top the sensor. The resistances collected from the sensor’s response to the masses were correlated to the pressure those masses applied, and a calibration curve was constructed; the resistance data read from the micro controller can now be directly related to the applied pressure on the strut. The specific sensor used in the gripper is the Interlink Electronics 30-81794 Model 402 FSR capable of sensing 0.2–20 N of force. Since its active sensing area is 14.68 mm^2^, its pressure sensing range is 13.62–1,362 Pa. For an FSR calibrated across its entire pressure sensing range, its resolution is 1.317 Pa when read by an Arduino. The analog pressure gauge is used during testing and prototyping for information about the air pressure inside the gripper as it inflates.

Lastly, negative temperature coefficient (NTC) thermistors measure the temperature of TCAMs during actuation to provide secondary feedback and prevent system damage. NTC thermistors are semiconductors whose response to temperature is controlled by the ratio of their composite materials. An NTC thermistor’s resistance decreases nonlinearly as temperature increases. This resistance can be converted to a temperature using the Steinhart-Hart equation (Steinhart and Hart, 1968), which models the resistance of a semiconductor at different temperatures. The equation requires three calibration constants calculated by measuring a thermistor’s resistance at a low, medium, and high temperature. To calibrate individual sensors, thermistor resistance values are measured alongside a previously calibrated thermocouple’s temperature output as they are heated from room temperature to 150°C, and 300°C to cover the thermistor’s full sensing range. The sensor’s resistance could then be read with an Arduino and graphed using the Arduino serial plotter. The specific sensors used were Amphenol Advanced Sensors TG-250-J-34-G-B-NR thermistors capable of sensing from −40°C–300°C. For a thermistor calibrated from 20°C to 300°C, its resolution is 0.2734°C when read by an Arduino. This range is well suited to cover the operational temperatures of TCAMs.

## 3 Pressure system

Applying desired pressure to the reversible adhesive at its point of contact is key to the presented gripper’s function. Design inspiration for the gripper’s pressure system is taken from commonly seen Velcro locked blood pressure pumps. This system relies on a fluid input to a chamber in order to inflate it and direct a pressure inward toward it is target, a bonding point between two struts. The Velcro locking mechanism is replaced in this case by an electromagnet positioned as illustrated in Figure 1, which keeps the system locked as pressure is applied. Five different iterations of the pressure silicone body are molded; the variations include differences in material, pneumatic channel geometry, and the use of embedded inelastic material. All iterations use one of two different silicone materials, EcoFlex 35 Fast, and EcoFlex 50.

**FIGURE 3:**
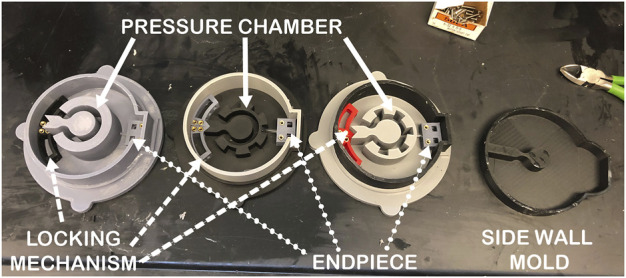
3D printed silicone gripper mold pieces for pressure application. Each have an outer diameter of 10.16 cm, an inner diameter of 2.54 cm, a chamber thickness of 1.27 cm, side wall thicknesses of 1.91 cm, and the same hard hybrid components (also 3D printed). The differences are in the geometry of the pressure chamber, with the far left having a “C” shape, center having outward expansion chambers, and the right most having inward expansion chambers.

Figure 3 displays the three molds that created these iterations. The geometries of the pneumatic channel can be seen inside the outer wall of the molds. These geometries are (from left to right in the Figure) a complete “C” shape, a “C” shape with outward expansion chambers, and a “C” shape with inward expansion chambers. One gripper iteration, which uses the “C” shaped pneumatic channel, has embedded fiber glass as an inextensible layer between the side walls, and the edge of the outer diameter. Three of the presented tests directly compare the output potential of the three proposed inner geometries by fixing the material, sidewall thickness, and chamber thickness of the tested prototypes in order to choose an optimal geometry. The inner diameter of each gripper is 2.54 cm, the outer diameter is 10.16 cm, and thickness of each of the grippers is 5.08 cm. These dimensions are chosen in accordance with a small scale test rig constructed for the validation of reversible adhesives. In the interest of mitigating difficulties in test assembly, the working fluid chosen for system tests is air. The findings of these small scale tests will inform future testing on larger scales. It is assumed that despite changes in scale and from air to incompressible fluid, these tests are a good indicator of design functionality and feasibility, but they do not fully describe the systems capabilities.

The amount of pressure applied to the strut can be determined using a testing rig that integrates 3D printed struts and sensors. A control board, which consists of a microcontroller, potentiometer knobs, solenoid valves, and pneumatic tubes, is used to pump air into the pressure channel within the silicone body of the gripper. As the air inflates the channel, the FSR and analog pressure gauge will determine the pressure applied to the strut and the pressure within the chamber, respectively. Thus, a relationship between pressure input to the system and pressure applied to the strut can be determined. This test can be conducted until the grippers experiences a material failure, which results in the maximum pressure applied to the strut being recorded by the microcontroller. Table 1 shows the maximum pressure applied by each iteration. Overall, the best performing gripper is the iteration with the fiber glass embedded inelastic layer, which can apply a 0.15 MPa pressure to the strut. In the future, inextensible layers should undergo further testing and iteration; these layers can aid in the maximum pressure applied, and the overall strength, and durability of the gripper. Preliminary results from this test also suggest that the simple “C” shaped geometry may out perform the other designs. In addition to this, the thickness of the gripper will be expanded to allow for a longer range of compliance; these tests represent a small cross section of this expanded gripper and strength, durability, and the pressure able to be applied will be positively affected by this change.

**TABLE 1:** Maximum applied pressure test results for gripper iterations.

Material	Side wall (cm)	Chamber thickness (cm)	Geometry	P (KPa)
EcoFlex 35	0.635	2.540	Inward	120
EcoFlex 50	1.905	1.270	Inward	35
EcoFlex 50	1.905	1.270	Outward	51
EcoFlex 50	1.905	1.270	“C″	71
EcoFlex 50 w/fiber glass	1.905	1.270	“C″	150

## 4 Induction system

### 4.1 System sizing test

Induction allows for an exchange of energy without the need for direct contact, meaning the heating element of the gripper will not need to touch the adhesive. By running an alternating current (AC) through a wire, the changing magnetic field can induce eddy currents within nearby metallic objects. The metallic resistance of the object causes it to increase its temperature when subjected to the eddy current. This means adhesive can be heated indirectly when applied to a metal sleeve clamped around the two strut elements that will be bonded together. For testing, pressure is applied to the sleeve through threaded aluminum clamps instead of the gripper and heat is applied through means of induction to demonstrate the capability of an induction coil to bond the reversible adhesive.

In order to achieve a successful bond or debond, the adhesive needs to be maintained at a minimum of 340°C with a pressure of 0.5 MPa. To reach this, a handheld inductor, operating at 120 V AC, 50–60 Hz, and a bendable induction coil, measuring 1 m total, are considered for testing purposes. The handheld inductor approximates the power source available for the gripper to use, and the bendable coil is representative of a flexible material that can potentially be implemented into our soft system. To validate these components, an induction test is performed to show that the adhesive can reach 340°C. The test set up uses three thermocouple sensors; one to measure the air temperature inside of the hollow titanium strut, and the other two to be attached between the sleeve and strut. The bendable copper coil is wrapped around the two struts with a helical shape, completing four turns.

**FIGURE 4:**
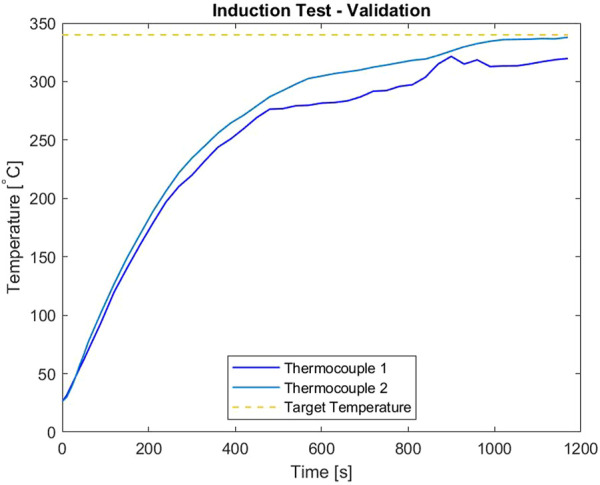
Initial Induction test results.

Over the course of approximately 1,200 s (20 min), the struts and metallic sleeve heated up to the desired 340°C. The collection of data from the thermocouples is illustrated in Figure 4, where the temperature of the sleeve and struts are Thermocouple 1 and Thermocouple 2, respectively. The experiment was concluded after the sleeve and struts reached the 340°C threshold. This validation experiment confirmed that going forward, the power output given by the handheld inductor along with the bendable coil could be used for further testing to reach the target temperature of 340°C.

### 4.2 Coil configuration experiments

We used an FLIR infrared camera to measure the temperature at distinct points of the induction heating experiments. Utilizing this feature, a nodal heat map can be created which allows for different iterations of coil geometries to be directly compared with one another. This map will determine which areas heat up the quickest for a given coil geometry and where the most effective placement of the workpiece is within that geometry. The test set up to create the nodal heat map consists of a non-metal base not be affected by induction and steel nails placed radially on that base. The nails serve as the nodes of heat map to be generated and their temperature is measured with the infrared camera. In addition to being affected by induction, the nails are also used to help guide the bendable coil into the specific geometries. An insulation blanket is used to avoid direct contact between the coil and nails when arranging these geometries. Without the insulation, the nails directly in contact with the bendable coil will increase in temperature drastically and damage the coil.

The geometries which are tested with the 1 m bendable coil include a single radial wrap, a double radial wrap, and a “C” shape (where the coil runs one direction, then bends and runs back the other direction). Different distances from the center radial nail are iterated for each of these geometries. Each test lasted for 50 s, and infrared photographs were taken at 10 s intervals. The nodal points increased in temperature with the single and double radial wrap, with the nails closest to the coil being affected the most. The nodal points did not experience a change in temperature with the “C” shape coil. This short coming is due to the magnetic fields of the wires canceling out from opposing directions they are traveling noting that only a single back and forth for the wire was used due to the length of the bendable coil. By increasing the total length of the bendable coil and creating a spiral shape, this problem can be fixed. Based on these results, a spiral square bendable coil is chosen for use in further testing. Not only is it believed that this design would work most efficiently, but it would also integrate best with the current design of the gripper.

## 5 Gripper deflection and actuation system

The actuation of this soft robotic gripper is to be performed by a biologically inspired muscle-tendon mechanism where CF/SR TCAMs supply tension to the system, pulling a tendon embedded in the gripper and opening the device. The challenge of implementing this or any actuation methodology stems from the system’s high operational temperature which is inhospitable to sensory equipment and to the thermally actuated muscles. This can be mitigated by holding the TCAMs away from the gripper in a wrist, transferring their applied tension on the system through attached tendons. However, sensory issues remain as flex or pressure sensors embedded in the gripper are likely to be damaged during operation. From a controls perspective, the lack of feedback is unacceptable for the implementation of a robust control methodology, so sensory systems must also be moved to the wrist. Load cells sensing tendon tension as it is applied to the gripper can be used in conjunction with the PCC modeling techniques to generate model-based feedback and allow for controller derivation.

### 5.1 Piecewise constant curvature modeling for feedback

The PCC model relies on the assumption that a thin flexible body can be discretized into inextensible sections which trace the arcs of circles with time dependent radii. Applying this to the gripper, a two-dimensional constant curvature model can capture the thin cross section of the gripper and it can be assumed that deflection is homogeneously distributed along its width. Additional simplification can be made by assuming that a single constant curvature segment will adequately capture one-half of the gripper in the cross section and that the tendon tension will be evenly distributed between the device’s two sides.

The following is a description of constant curvature kinematic and dynamic models for one side of the gripper, developed from the guidelines of surveys of the field Della Santina et al. (2021); Webster and Jones (2010). First, it is assumed that the centerline of the gripper’s cross-section behaves as a deformable rod in a two dimensional plane. The shape of this rod is given by the vector

$$h\left(s,q\left(t\right)\right)=L{\left[\frac{sin\left(sq\left(t\right)\right)}{q\left(t\right)},\frac{1-cos\left(sq\left(t\right)\right)}{q\left(t\right)},\frac{sq\left(t\right)}{L}\right]}^{⊤},$$ (4)

where *L* is the length of the modeled side of the gripper, *s* is the position along the length of the one dimensional rod and *q*(*t*) is the angle describing the deflection of the rod relative to a unbent configuration. In the case of the gripper, the initial kinematic conditions of the rod are given by *q*(0) = *π* radians. The Jacobian giving the relationship $\dot{h}\left(s,q\left(t\right)\right)=J\left(s,q\left(t\right)\right)\dot{q}\left(t\right)$ can be solved as

$$J\left(s,q\left(t\right)\right)=\frac{\partial h\left(s,q\left(t\right)\right)}{\partial q\left(t\right)}$$

The dynamics for a single segment are given by the ordinary differential equation,

$$M\left(q\right)\ddot{q}+C\left(q,\dot{q}\right)\dot{q}+G\left(q\right)+D\left(q\right)\dot{q}+K\left(q\right)=A\left(q\right)\tau .$$ (5)

In this simplified case, the rod is assumed to have a thin cross section and uniform distribution of mass leading to an inertia scalar

$$M\left(q\right)=\frac{mL^{2}}{3}\left(\frac{q^{3}+6q-12sin\left(q\right)+6qcos\left(q\right)}{q^{5}}\right).$$

Coriolis and centrifugal forces are given by $C\left(q,\dot{q}\right)=\frac{1}{2}\left(\frac{dM\left(q\right)}{dt}\right)$ , gravitational torque is given as

$$G\left(q,\phi \right)=-mg{\left(\int_{0}^{1}J\left(s,q\right)ds\right)}^{⊤}\left[\begin{array}{c} cos\left(\phi \right)\\ sin\left(\phi \right)\\ 0 \end{array}\right],$$

and the stiffness and damping of the rod is assumed to be uniform such that *K* and *D* are constant. The actuation matrix for the modeled gripper’s configuration is given by *A*(*q*) = *J* (1,*q*)^*⊤*^. If the input is assumed to be a pure torque, *A*(*q*) = 1.

### 5.2 Small scale validation

**FIGURE 5:**
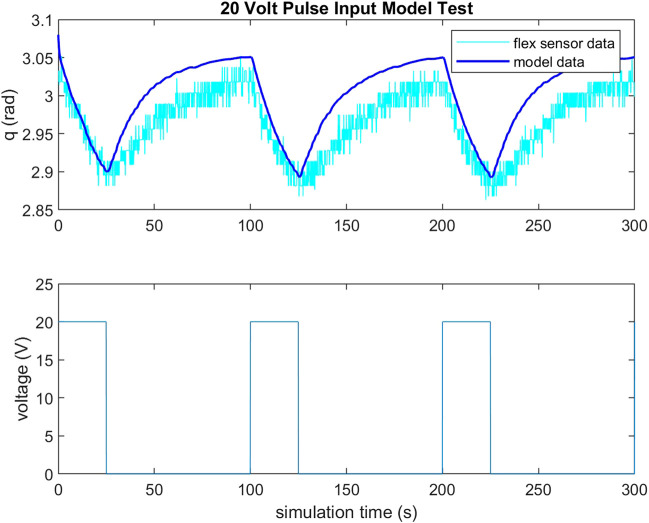
Flex sensor data compared to PCC model output with feedback from load cell in gripper tendons for 20 V pulse input to TCAMs. Note that the test is run in simulink, so simulation time does not exactly equate to seconds.

**FIGURE 6:**
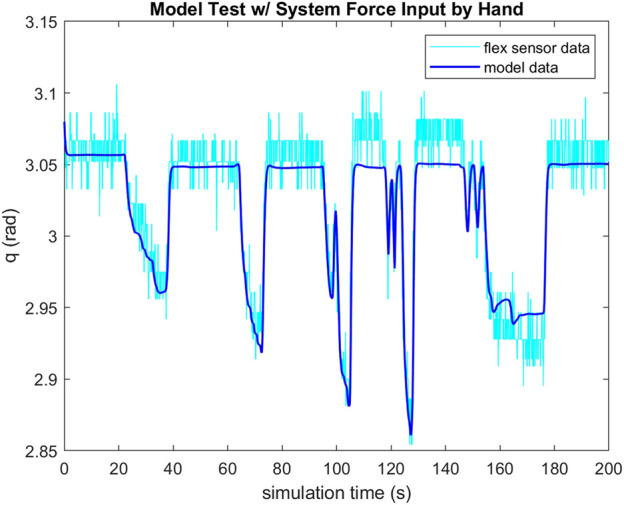
Flex sensor data compared to PCC model output with feedback from load cell in gripper tendons with tension input to the system by hand.

**FIGURE 7:**
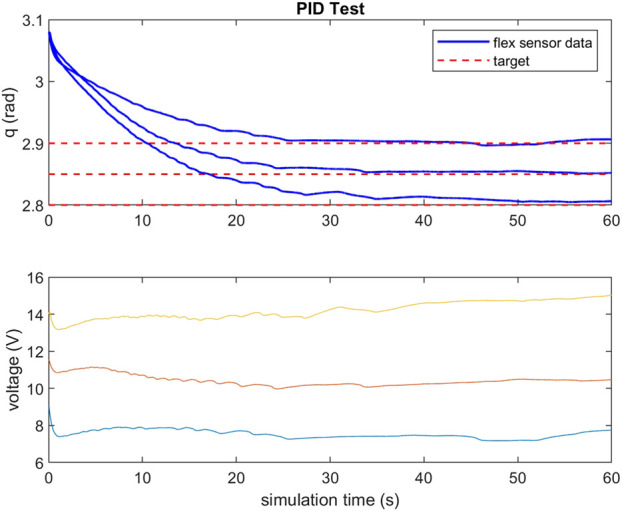
PID test for targeted deflections and required voltages.

**FIGURE 8:**
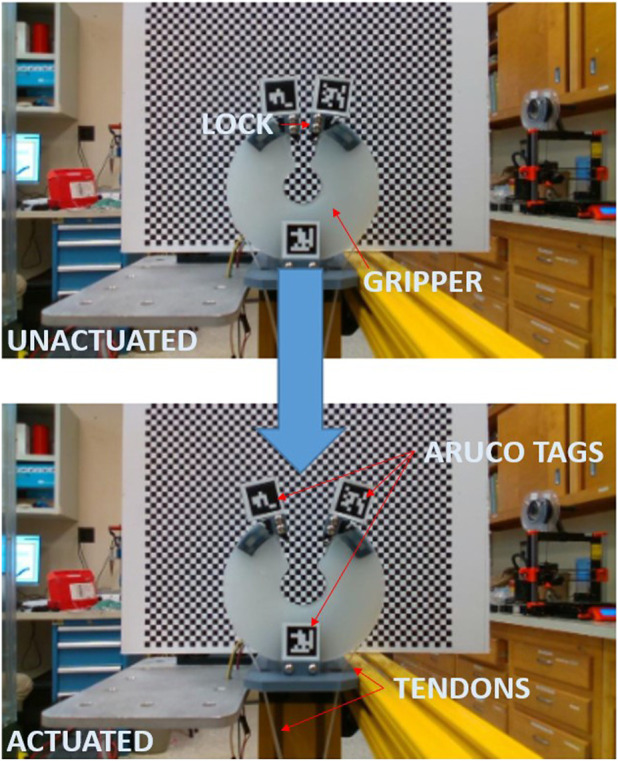
Small scale test gripper in closed and partially open state actuated by TCAMs.

To validate the feasibility of the gripper’s actuation system, a small scale gripper was produced with the assumption that its capabilities would scale to larger systems. Observing Equation 2, TCAM temperature regulates their stiffness and Equation 3 implies that voltage is a suitable system input to control a TCAM’s force output. For the purposes of this study, a simple PID controller is implemented to regulate the system input voltage in order to reach a desired deflection *q* in the gripper based on feedback from the PCC model described by Equations 4, 5. A load cell informs the model of the input tension to the system. To validate this feedback method, a test set up is constructed wherein a test gripper has two flex sensors embedded along the center-line of its right arm. It is assumed that the tendon force is equally distributed between the two-halves of the gripper and that the deflection of one-half is matched by the other. Feedback from these flex sensors is used as a check of the PCC model’s ability to capture the deflection of the gripper. Figures 5, 6 demonstrate the ability of the presented PCC model to capture the position of the gripper given by the embedded flex sensors. In Figure 5, a 20 V pulse is input to the system and flex sensor data is compared to the model output from tension feedback. A close match is observed while the system is under increasing tension, giving the model credibility for estimating a desired deflection while under load. In Figure 6, a test is performed where tension is given to the system by hand, and again we see good agreement between the model and flex sensor data. Figure 7 demonstrates that the PID controller implemented for the task of deflection regulation is capable of imposing a desired state on our system. A visual representation of this actuation can be seen in Figure 8. This validation is focused on the reliability of PCC modeling techniques for system pose feedback in the absences of more direct measurements and it should be noted that more specialized control methodologies like the one presented in Hammond et al. (2021) could be used to better regulate the behavior of the system and ensure safe and desired behavior from TCAM actuation.

## 6 Simulation

We investigate the fidelity of modeling the soft gripper in the open-source simulation software Gazebo. A key advantage of Gazebo is its compatibility with the Robot Operating System (ROS 2). Using Gazebo and ROS 2 in conjunction, we can simulate the end effector, apply forces to cause actuation, and record curvature data in real-time to compare against hardware test data. Analyzing the accuracy of our Gazebo simulation model against the real hardware testing data will provide a higher degree of confidence in modeling future soft robotic systems in the mod/sim environment. This will be especially important when it comes to modeling more complex, multi-agent ISAM systems that will incorporate soft robotic end effectors.

In ROS 2, robot models are defined using a universal Robot Description File (URDF) that organizes the robot into geometric links connected by joints. When a new link is added to the model, it is connected to a “parent” link by a joint that determines how the new “child” link is allowed to move relative to its parent. Gazebo converts the URDF file to the Simulation Description Format (SDF) when it receives the file from ROS because Gazebo is not compatible with URDF. When building a robot in URDF for ROS 2 and Gazebo, only the joint types supported by both URDF *and* SDF are allowed: revolute (limited rotation about one axis), continuous (unlimited rotation about one axis), prismatic (limited translation along one axis), and fixed (all motion constrained) SDFormat (2020), URDF (2022). In addition, links in URDF are constrained to be rigid; they do not bend or stretch as soft robots do. With these challenges in mind, our solution when modeling the flexible, continuous end effector is to divide it into a series of discrete, rigid links connected by revolute joints, as inspired by the work of Chen et al. (2018). By giving these joints spring properties, we can approximate the real gripper’s actuation behavior.

### 6.1 Designing the simulated gripper

**FIGURE 9:**
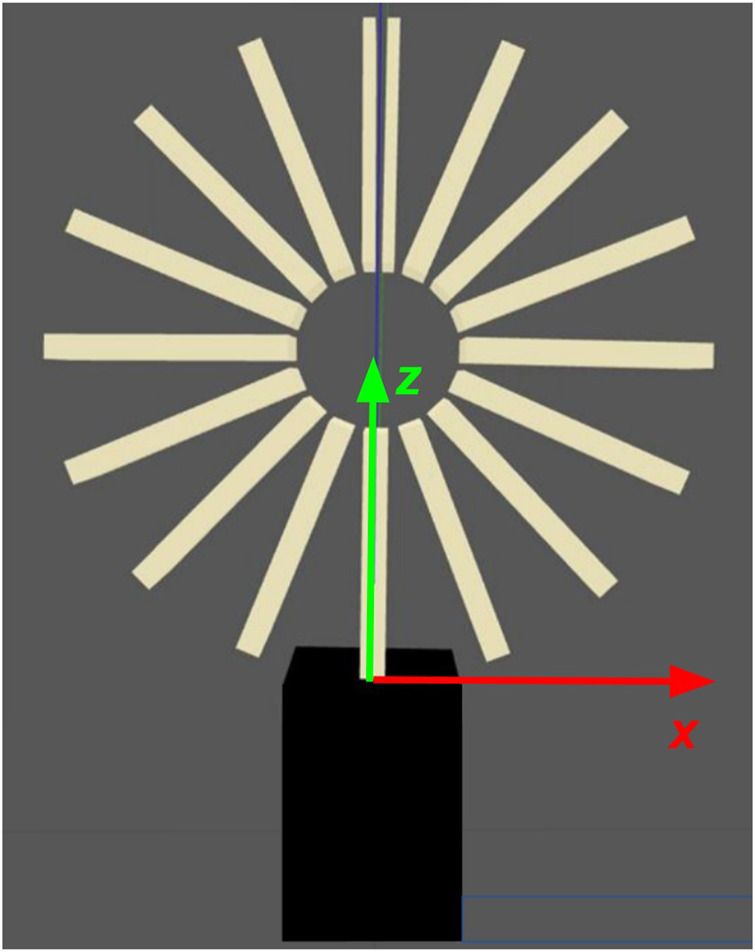
Gazebo gripper model in neutral state.

The gripper model is divided into two curved arms, each composed of eight “box” links. A stationary link at the base of the robot anchors the gripper to a platform and acts as the first parent for the set of links in each arm. When the end effector is in a neutral state where gravity is the only force present, the arms curl up into a closed circular shape that replicates the real end effector, as illustrated in Figure 9. When modeling the soft gripper, there are several parameters to consider: the total number of links in the model, the mass of each link, the dimensions of the links (thickness, height, and width), the separation distance between links, and the spring properties of the joints (the spring constant and the spring reference angle). All of these parameters impact the shape of the soft gripper model when it is in static equilibrium. Since dynamic modeling is not considered, the spring damping constant of the Gazebo gripper’s joints is assigned an arbitrarily large value to eliminate oscillations during the testing procedure.

First, we choose a set number of links *N* that we anticipate will give a reasonable approximation of the real gripper. For a total mass *M* of the real gripper, the mass of each link in the model is *m* = *M*/*N*, assuming there is an even distribution of mass from a perfectly poured silicone mold. We choose *N* = 16 and measure *M* = 0.2238 kg from the real soft gripper used in experimentation, so *m* = 0.014 kg/link. It is important to note that in the model, the final link on each arm has half the thickness of all the other links so that when the gripper is closed the model is symmetrical across the vertical axis. When calculating the mass per link, these two half-sized links each only count as half a link. Therefore, while there are 17 links in the model (eight on both sides and one in the center), we use *N* = 16 when determining *m*.

**FIGURE 10:**
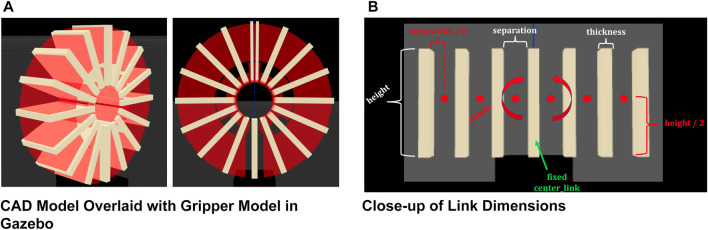
Simulated gripper design. **(A)** CAD Model Overlaid with Gripper Model in Gazebo. **(B)** Close-up of Link Dimensions.

Next, a CAD file of the gripper that was used to create the silicone mold is loaded into Gazebo as a semi-transparent link with no physical tangibility and overlaid with the gripper model, as shown in Figure 10A. This CAD model serves as a reference for the shape of the real gripper when it is in a neutral state. Keeping the number of links and the mass per link constant, the rest of the parameters are manually adjusted until the soft gripper model matches the shape of the CAD model. The spring reference constant defines the neutral rotation angle of each revolute joint, and the spring stiffness constant, given by Hooke’s Law for torsion spring, defines how well the joints resist a change in angle due to an external torque. These two values are manually adjusted through visual curve fitting until the neutral curved shape of the soft gripper model approximately matches the circular shape of the CAD model. For simplicity, all of the joints in the model are assigned the same spring values. The spring reference is defined as 0.390335 radians, and the stiffness constant is initially defined as 2 Nm/radian.

Finally, with the curved shape of the soft gripper model matching the shape of the CAD model, we adjust the separation distance between links and the thickness of the links to prevent links from overlapping with each other and disturbing the model’s stability. Figure 10B shows how the dimensions of links are defined. The image shows a close-up view of the base of the gripper when the arms are fully stretched out horizontally. Each joint in the model is located at the midpoint between links. The height and width of the gripper are determined by the dimensions of the CAD model, but since they have no impact on the curvature of the model, they are only defined for aesthetics. Figure 10A shows the final design of the gripper, overlaid with the CAD model. The separation distance is 9 mm, and the thickness is 3.9 mm.

By modifying these parameters of the gripper model, we can approximate the shape of the real soft gripper when the TCAMs are not activated. To replicate the static equilibrium state of the real gripper for different deflection angles, we modify the spring stiffness constant through visual curve fitting during the testing procedure.

### 6.2 Testing the simulation model

**FIGURE 11:**
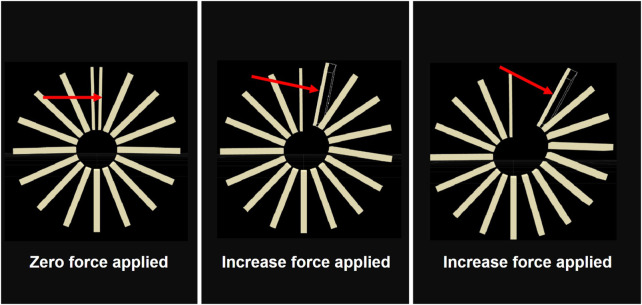
Force applied to one arm of the model in gazebo.

When performing actuation tests on the real gripper, a controller is used to adjust the voltage applied to the TCAMs and actuate the gripper to a desired deflection angle. In the equilibrium state when the gripper reaches a desired angle, the tension in the TCAMs is measured by a force sensor. To compare the actuation of the Gazebo gripper with the real gripper, the tension measured in the TCAMs is replicated in Gazebo by applying the force perpendicularly to the last link on each gripper arm. This is accomplished using the gazebo_ros_force_system plugin. Figure 11 illustrates where the force is applied to the model. When the gripper reaches static equilibrium, the rotation angle of each joint is recorded by a gazebo_ros_joint_state_publisher node. Then, the coordinate (x, z) positions of each joint relative to the base of the gripper are calculated using forward kinematics. This process is repeated for different equilibrium tension measured in the TCAMs, and then the curvature data is compared with the data collected for the real gripper.

Using the deflection angle q measured by the flex sensors and PCC kinematics as presented in Webster and Jones (2010), we can report the (x, z) coordinate of a position s along the real gripper arm center-line relative to the origin of the gripper. We choose values of s that correspond with the joint locations along the Gazebo gripper, and this gives us a set of (x, z) coordinates along the real gripper that we can compare with the joint positions on the Gazebo gripper for each deflection angle. By graphing both sets of joint coordinates for every deflection angle on an *x*-*z* axis, we can assess how well the Gazebo model follows the real gripper for different angles. We then modified the spring stiffness constant and repeated the Gazebo testing and assessment procedure to minimize the error between Gazebo joint positions and real joint positions.

For additional model validation, we attach three Aruco markers to different points on the simulated and real gripper and use optical sensors to capture images of the grippers when they are in static equilibrium. In the real hardware test, we mount an Intel RealSense Camera D435i to the testing rig. In Gazebo we create an optical camera link using the gazebo_ros_camera_plugin and position it relative to the gripper based on measurements from the real hardware test. Images captured by the cameras are run through an Aruco pose estimation program that generates a transformation matrix mapping each Aruco marker in the image to the location of the camera. We intend to compare the positions and orientations of the corresponding markers Gazebo and real images as an additional layer of model testing. However, before this data is analyzed, steps must be taken in the future to quantify the error in pose estimation measurements in Gazebo.

### 6.3 Simulation results

**FIGURE 12:**
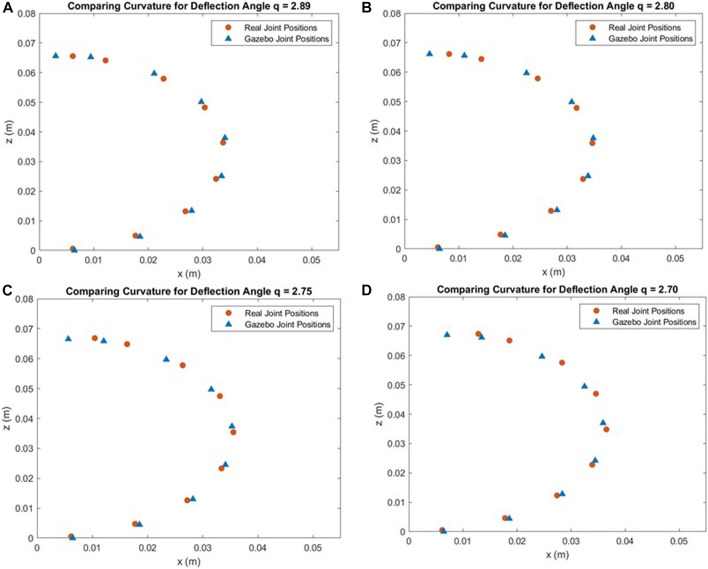
Comparing Gazebo and Real Data for Different Deflection Angles (q) **(A)** q = 2.89. **(B)** q = 2.80 **(C)** q = 2.75. **(D)** q = 2.70.

We found that a spring stiffness constant of 5 Nm/radian reduces the magnitude of difference between Gazebo and real coordinate values to an average of less than 5 mm. Figures 12A–D plot the collected Gazebo and real joint positions for four different deflection angles measured in the real gripper. In each figure, the (*x*, *z*) coordinates of the joint states of the simulated end effector and the real end effector are plotted on the same axes.

**FIGURE 13:**
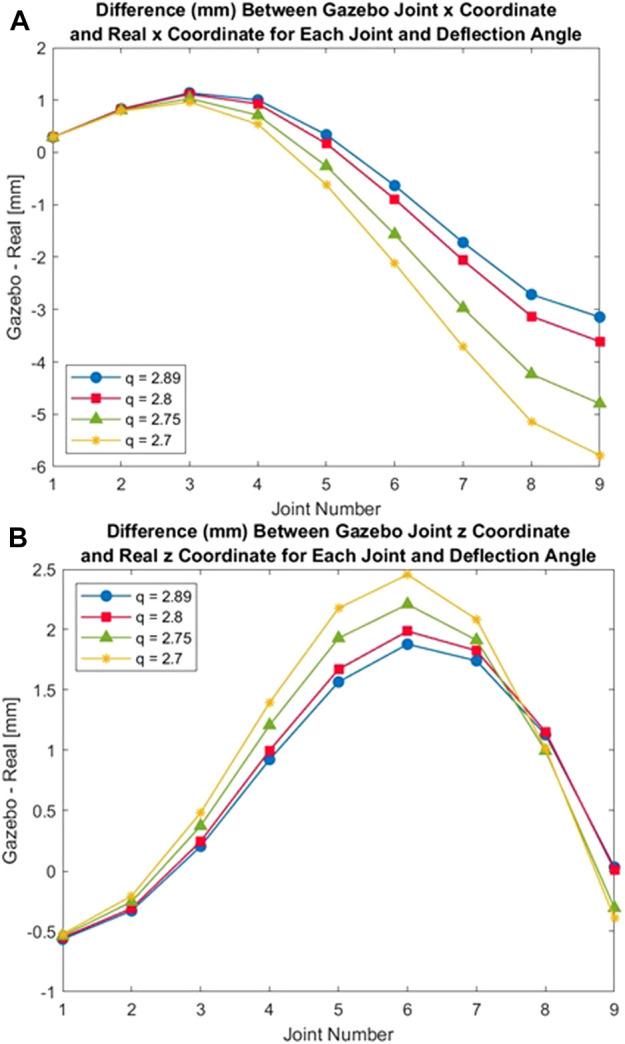
Error in Gazebo Joint Coordinate Positions **(A)** Error in Gazebo x-Coordinate Position. **(B)** Error in Gazebo z-Coordinate Position.

Figures 13A, B show the differences between the Gazebo gripper and real gripper for x and z coordinates of each joint. For the x coordinates in Figure 13A, the magnitude of the error is low for the first three joints (staying under 1 mm of difference), and the error is consistent between deflection angle trials. Between joints 5 and 9, however, the magnitude of the error increases for each subsequent joint to a maximum of 5.78 mm in joint 9 for *q* = 2.7. The error between the simulated and real joint locations also grows as more force is applied to the end effectors (larger deflection *q*). This trend is also apparent in Figure 13B that shows the difference between real and simulated z coordinates. For almost every joint along the gripper arm, a larger force applied to the end effectors results in a larger error between the real and simulated positions, particularly along the *x*-axis. The magnitude of the error peaks at 2.46 mm at joint 6 for *q* = 2.7.

These results imply that the accuracy of the Gazebo model often decreases 1) moving farther from the stationary anchor and 2) as more force is applied to the gripper (resulting in a larger deflection). One contributing factor is that, by design, the Gazebo simulation model approximates a continuous, flexible gripper as a series of discrete links. The model has far fewer degrees of freedom than the real gripper (near infinite), and the model’s ability to accurately replicate every position of the real hardware, therefore, is limited. In the future, the Gazebo model could be divided into a larger number of links to observe the impact on model accuracy. This would require re-tuning all of the other model parameters to ensure that the model still matched the real gripper when in a neutral state.

In addition, the accuracy of the hardware sensor measurements could also increase the error between real and simulated gripper joint positions. First, the curvature of the real gripper is recorded by flex sensors that depend on an assumption that the curve it measures is constant. As described in Section 5.1, we assume that the end effector exhibits PCC, but if this assumption is inaccurate, then the accuracy is limited. Second, we assume that the force measured by the force sensor close to the TCAMs has minimal loss when transmitted to the end of each gripper arm through tendons. In the Gazebo simulation, the force recorded by the real force sensor is directly applied to the last link in both gripper arms. Therefore, if there is a significant loss between the force measured at the TCAMs and the actual force applied to the real end effector, then this could limit the accuracy of the Gazebo simulation results.

Future work for simulating the soft gripper could include decreasing the uncertainty in the hardware sensor measurements, performing a dynamic system analysis, and simulating the gripper using multi-physics finite elements modeling. Additionally, a more sophisticated method of determining the Gazebo model parameters is needed for better fitting of the simulation model of the gripper to the real gripper. The spring constants could be calculated directly if the spring characteristics of the soft gripper material were thoroughly analyzed. Additionally, a mathematical relationship could be devised that relates the spring variables to coordinate positions of the model using known information about the mass of each link. This would improve the process of choosing model parameters.

## 7 Conclusion

From vine robots developed by Stanford University to soft robotic grippers developed by On Robot, soft material robotics has many uses for terrestrial applications. These applications range from search and rescue though hazardous zones to fragile object handling and food preparation. For space applications, elastomers are used for connection points on rocket systems, soft materials are incorporated into space suits, and inflatables can be integrated into spacecraft like the Bigelow Aerospace’s Bigelow Expandable Activity Module (BEAM); however, soft material robotics is still considered a nascent field and in a low Technology Readiness Level (TRL) category. To increase that TRL, NASA LaRC researchers are working with state-of-the-art materials and robotic systems to continue studying the applications of soft material systems for in-space assembly operations.

In this paper, early work has been presented on a soft robotic gripper designed for conical strut joining using reversible adhesives. We have shown one possible design, Figure 1, that encompasses the key elements needed for the specific task of conforming to conical struts. This design contains the gripping system, actuated by TCAM artificial muscles, the pressure system, pneumatically or hydraulically supplied for bonding/de-bonding, and the heating system using an induction system. From this design, we’ve shown preliminary results on TCAM actuation, pressure system, heating system, and modeling and simulation of the hybrid soft gripper linked to the control methodology presented. We’ve also described the bench top sensing system used for data gathering and the feedback mechanism for our model validation in Gazebo.

Developing and testing this unique end effector will help increase reliability during assembly operations on large scale in-space structures using non-traditional strut configurations. Unit testing with the TCAM actuation, pressure system, induction, control methodology on the bench were all conducted to better understand the operational nature of the design, providing the team insight on needed modifications for an actualized and fully integrated robotic gripper. Modeling and simulation tests were conducted in parallel with hardware and software development to verify the design of the robotic gripper prototype and understand the limitations of the Gazebo/ROS 2 environment for modeling future soft robotic systems.

The procedures and results from this project will be used for the continued development of the hybrid soft material gripper and other end-effector work. Due to the inherent compliance found with soft materials, this end effector design may have broader usage for component manipulation and bonding requirements for future in-space assembly and disassembly needs.

## Data Availability

The raw data supporting the conclusions of this article will be made available by the authors, without undue reservation.
